# A prospective cohort study of the association between drinking water arsenic exposure and self-reported maternal health symptoms during pregnancy in Bangladesh

**DOI:** 10.1186/1476-069X-13-29

**Published:** 2014-04-16

**Authors:** Molly L Kile, Ema G Rodrigues, Maitreyi Mazumdar, Christine B Dobson, Nancy Diao, Mostofa Golam, Quazi Quamruzzaman, Mahmudar Rahman, David C Christiani

**Affiliations:** 1 Department of Public Health, College of Public Health and Human Sciences, Oregon State University, 15 Milam, Corvallis, OR 97331, USA; 2 Department of Environmental Health, Harvard School of Public Health, Boston, MA, USA; 3 Dhaka Community Hospital Trust, Dhaka, Bangladesh

**Keywords:** Arsenic, Maternal health, Nausea, Vomiting, Cramping, Environmental health, Reproductive health

## Abstract

**Background:**

Arsenic, a common groundwater pollutant, is associated with adverse reproductive health but few studies have examined its effect on maternal health.

**Methods:**

A prospective cohort was recruited in Bangladesh from 2008–2011 (N = 1,458). At enrollment (<16 weeks gestational age [WGA]), arsenic was measured in personal drinking water using inductively-coupled plasma mass spectrometry. Questionnaires collected health data at enrollment, at 28 WGA, and within one month of delivery. Adjusted odds ratios (aORs) and 95% confidence intervals (95% CI) for self-reported health symptoms were estimated for each arsenic quartile using logistic regression.

**Results:**

Overall, the mean concentration of arsenic was 38 μg/L (Standard deviation, 92.7 μg/L). A total of 795 women reported one or more of the following symptoms during pregnancy (cold/flu/infection, nausea/vomiting, abdominal cramping, headache, vaginal bleeding, or swollen ankles). Compared to participants exposed to the lowest quartile of arsenic (≤0.9 μg/L), the aOR for reporting any symptom during pregnancy was 0.62 (95% CI = 0.44-0.88) in the second quartile, 1.83 (95% CI = 1.25-2.69) in the third quartile, and 2.11 (95% CI = 1.42-3.13) in the fourth quartile where the mean arsenic concentration in each quartile was 1.5 μg/L, 12.0 μg/L and 144.7 μg/L, respectively. Upon examining individual symptoms, only nausea/vomiting and abdominal cramping showed consistent associations with arsenic exposure. The odds of self-reported nausea/vomiting was 0.98 (95% CI: 0.68, 1.41), 1.52 (95% CI: 1.05, 2.18), and 1.81 (95% CI: 1.26, 2.60) in the second, third and fourth quartile of arsenic relative to the lowest quartile after adjusting for age, body mass index, second-hand tobacco smoke exposure, educational status, parity, anemia, ferritin, medication usage, type of sanitation at home, and household income. A positive trend was also observed for abdominal cramping (P for trend <0.0001). A marginal negative association was observed between arsenic quartiles and odds of self-reported cold/flu/infection (P for trend = 0.08). No association was observed between arsenic and self-reported headache (P for trend = 0.19).

**Conclusion:**

Moderate exposure to arsenic contaminated drinking water early in pregnancy was associated with increased odds of experiencing nausea/vomiting and abdominal cramping. Preventing exposure to arsenic contaminated drinking water during pregnancy could improve maternal health.

## Background

Inorganic arsenic is a ubiquitous environmental pollutant. People can be exposed to arsenic from occupational activities, eating contaminated foods, and from industrial sources [1]. The most common route of exposure, however, is from drinking groundwater that is contaminated with naturally occurring arsenic [2,3]. In the United States, a survey of all major aquifers estimated that 7% of households equating to approximately 4.2 million people are exposed to arsenic concentrations that exceed the United States Environmental Protection Agency’s maximum contaminant level of 10 μg/L [4]. However, arsenic-contaminated drinking water is a global health concern particularly for Bangladesh. This is because public health interventions designed to reduce waterborne disease switched the population’s source of drinking water from surface water to groundwater [5]. Unfortunately, the shallow groundwater aquifer under Bangladesh is contaminated with naturally-occurring arsenic [5]. As a result of this intervention, 46% of the population are exposed to arsenic concentrations above the World Health Organization’s recommended limit of 10 μg/L and 27% are exposed to concentrations above 50 μg/L which is the Bangladesh government’s drinking water recommendation (and previous U.S. limit) [6].

There is consistent evidence that chronic exposure to arsenic from drinking water increases the risk of skin lesions, cardiovascular disease, neuropathy, and type 2 diabetes and cancers of the skin, bladder, lung and kidney cancer, [7-14]. Data from epidemiological studies also show that chronic exposure to arsenic contaminated drinking water is associated with adverse reproductive health effects [15-19]. These reproductive health studies focused on the effect of arsenic on fetal loss, preterm birth, and/or birthweight. None have reported on the effect of chronic arsenic exposure on maternal health.

We decided, therefore, to examine the association between arsenic exposure and maternal health, as assessed by self-reported symptoms during pregnancy. We conducted this study in a large population-based prospective cohort of pregnant women recruited in Bangladesh. In this prospective reproductive health cohort, women were asked questions about their health and followed throughout the course of their pregnancy. We hypothesized that arsenic exposure measured in maternal drinking water at the time of enrollment into the cohort would be associated with increased self-reported symptoms of illness reported during the pregnancy.

## Methods

### Study area and subject selection

A prospective birth cohort was recruited in the Sirajdikhan and Pabna Sadar Upazilas of Bangladesh from 2008–2011. The objective of this cohort was to observe the effects of chronic low level arsenic exposure on reproductive outcomes. These Upazilas were selected for the study area because a national survey conducted by the British Geological Survey indicated that the average concentration of arsenic in the groundwater in these areas was more moderate than other regions in Bangladesh yet spanned a wide range of concentrations [6]. Additionally, Dhaka Community Hospital Trust (DCH) operates rural health clinics in these Upazilas that offer prenatal care and promote arsenic awareness by encouraging people to only drink water from wells that comply with the Bangladesh drinking water standard of ≤ 50 μg As/L. In addition to medical staff, each clinic has trained technicians who provided field testing of arsenic in well water upon request. DCH has also worked with villages in these Upazilas to implement safe water options [20].

Pregnant women were recruited by DCH-trained female community health care workers who live in the villages serviced by the clinics. Heath care workers were supplied with pregnancy test kits to identify potential participants. Women with a positive pregnancy test were told about the study and invited to the clinic to determine if they were eligible to participate in the study. Eligibility criteria included: ≥18 years of age, ultrasound confirmed singleton pregnancy of less than 16 weeks’ gestation, used a tubewell that supplied groundwater as their primary drinking water source, planned to live at their current residence for the duration of the pregnancy, planned to continue prenatal health care with DCH, and agreed to deliver at DCH or at home with a DCH-trained midwife.

The study involved four scheduled visits- at the time of enrollment (V1), at 28-weeks gestational age (V2), delivery (V3), and one month postpartum (V4). At V1 and V2, participants were asked to come to the clinic where they underwent a physical exam including an ultrasound and completed detailed questionnaires regarding their medical, pregnancy and drinking water histories. Participants also provided biological samples for medical tests and trace metal analysis. The medical questionnaires were also administered at V4. This analysis uses information collected in the medical questionnaire throughout the pregnancy (V1, V2, and V4). Cross-sectional analyses that used all data collected at V1 are included as Additional file 1: Supplemental Tables 1 and 2. As an incentive for participation, all women were provided with free prenatal care from DCH and prenatal vitamins that were replenished during monthly checkups in the participants’ homes.

Of the 1,458 participants with a confirmed single pregnancy that have data currently available for analysis, 196 (13.4%) were lost to follow up. This left 1,262 participants who completed all study visits. Complete information for all covariates was available for 1,165 of these participants. Informed consent was obtained from all participants before enrollment and prior to engaging in any study activities. During the consent procedure all participants were informed of the hazards posed by arsenic exposure using language that was consistent with the ongoing arsenic awareness campaigns (e.g. arsenic is a poison found in the groundwater that causes diseases of the skin and other parts of the body). Participants were also informed on the intention of this study which was to find out how arsenic affected the health of pregnant women and their infants. Participants were told about safe water options which could differ depending on their village circumstances [20]. Participants could request that their water be tested for arsenic using Merck (doubling method) field test kits by clinic staff [21]. For those participants that requested a field test, clinic staff made an appointment with the participant to come to their home to test their water. Since the consent process occurred during the first scheduled clinic visit, all arsenic field tests occurred after participants completed their initial questionnaires. Additionally, all participants were informed of the concentration of arsenic in their drinking water after it was analyzed by inductively coupled plasma mass spectrometry. This study was approved by the Human Research Committees at the Harvard School of Public Health, Dhaka Community Hospital and Oregon State University.

### Arsenic exposure

At the time of enrollment, a water sample was collected from the tubewell each participant identified as their primary source of drinking water. Briefly, water samples were collected in 50-ml polypropylene tubes (BD Falcon, BD Bioscience, Bedford, MA) and preserved with Reagent Grade nitric acid (Merck, Germany) to a pH <2. Samples were kept at room temperature prior to analysis by inductively coupled plasma-mass spectrometry following US EPA method 200.8 (Environmental Laboratory Services, North Syracuse, New York). The average percent recovery of As from PlasmaCAL multi-element Quality Control standard #1 solution (SCP Science, Canada) was 101% (range: 92%-110%). Of the 1237 samples included in this analysis, 252 (20.4%) had an arsenic concentration below the 1 μg As/L limit of detection (LOD). These samples were re-assigned half the value of the LOD for statistical analysis.

### Self-reported symptoms during pregnancy

The attending obstetrician asked the woman to recall symptoms three times during their current pregnancy (V1, V2, and V4). The questionnaire specifically asked whether the woman experienced any of the following groups of symptoms at least once during the time period covered by the study visit: cold, flu, rubella or other respiratory infections (yes/no); nausea and vomiting (yes/no); abdominal cramps (yes/no); vaginal bleeding (yes/no); swollen feet (yes/no); and severe headaches (yes/no).

### Covariates

At enrollment, information on women’s age, parity, education, socioeconomic status, access to sanitation, smoking history, medical and drinking water history, and information on the child’s father was collected. Educational attainment was collapsed into three categories (illiterate/able to write name, primary education, secondary education or higher). Parity was defined as the number of previous live births in multiparous women. Economic status was assessed directly by asking husbands their monthly income (less than 3000 taka, 3000–5000 taka, greater than 5000 taka, and refused to answer). Gestational age was determined by ultrasound measurements taken at the time of enrollment because few women could recall the date of their last menstrual period. Women’s weight and height were measured at enrollment and at each follow up visit. Body mass index (BMI) was calculated as weight divided by height squared (kilograms per square meter). Hemoglobin was estimated by mixing fresh whole blood with K3-EDTA prior to analysis by an automated hematology analyzer (Sysmex XS-800i, Sysmex India,). Anemia was defined as having a hemoglobin level ≤12 g/dL. Erythrocyte ferritin was measured in fresh blood and analyzed using an enzyme immunoassay IMx-Ferritin kit and an IMx analyzer. Severe ferritin deficiency was defined as ferritin <20 μg/L, mild deficiency was defined as ferritin within 20–40 μg/L, and no deficiency was defined as ferritin >40 μg/L.

### Statistical analysis

General descriptive statistics were calculated for select characteristics. Drinking water arsenic concentrations were transformed using the natural log and also categorized into quartiles. Chi-square tests and Wilcoxon rank sum tests were used to assess the association between outcomes and select characteristics. Logistic regression models were used to compute the relative odds and 95% confidence intervals of experiencing a symptom at least once during pregnancy for increasing quartiles of arsenic. Additionally, we constructed logistic regression models to compute the odds of self-reported symptoms for arsenic concentrations dichotomized at regulatory drinking water arsenic limits (below/above 10 μg/L and below/above 50 μg/L). We used a parsimonious approach towards model building and only included covariates in the multiple logistic regression models if they were associated with any self-reported symptom with an p < 0.05.

Since medication usage during pregnancy could influence symptoms experienced during pregnancy, we conducted a restricted analysis among women who reported that they did not take any herbal remedies or prescription during their pregnancy. Since the number of prior pregnancies is considered a risk factor for nausea and vomiting in pregnancy [22] we also examined potential effect modification by parity by including an interaction term between arsenic exposure and primiparous status.

## Results

We compared socio-demographic characteristics between the 1,262 women with complete follow up information with the 196 women who were lost to follow up. There was no significant difference in the age (22.8 vs 22.9 years, p-value = 0.51), ln drinking water arsenic (1.3 vs 1.7 μg/L, p-value 0.27), parity (0.7 vs 0.7, p-value = 0.76), BMI at the time of enrollment (20.8 vs 20.5, p-value = 0.42), or maternal education (p-value = 0.14) among participants who were lost to follow up and those who were included in this analysis. Clinic staff attempted to re-contact the women who were lost to follow up by going to their homes to determine why they did not complete the study protocols. None of these women could be re-contacted so reasons for lost to follow up could not be ascertained.

At the time of enrollment, the overall mean and median concentration of arsenic in participant’s drinking water was 38 μg/L (standard deviation, 92.7 μg/L) and 2 μg/L (interquartile range, 30 μg/L), respectively. Sixty-three percent of the women reported experiencing at least one health symptom during their pregnancy. The most common symptom reported by women was nausea/vomiting (39.2%) followed by abdominal cramping (37.1%), headache (32.7%), cold/flu/infection (18.0%), vaginal bleeding (1.0%), and swollen ankles (1.0%). Women who reported having any symptom during pregnancy were exposed to significantly higher concentrations of arsenic in their water compared to women who reported no symptoms (Table 1). Additionally, women who reported any symptom during pregnancy were more likely to be underweight, exposed to second hand tobacco smoke, have lower educational attainment, have anemia and lower ferritin status, lack sanitary latrines, take medication, and have lower household incomes compared to women who reported no adverse symptoms during pregnancy.

**Table 1:** Characteristics of 1,262 participants that reported experiencing any symptom during pregnancy versus no symptoms (2008–2010)

	**Reporting any symptom**		
	**No *(n = 467)***	**Yes *(n = 795)***	**p-value**
**Mean (SD), arsenic at enrollment (μg/L)**	20.9 (70.8)	50.0 (104.1)	*<0.0001*
**Median (IQR), arsenic at enrollment (μg/L)**	1.6 (0.8, 5.2)	7.0 (1.1, 58.0)	*<0.0001*^*a*^
**Mean (SD) age at enrollment (yrs)**	22.6 (4.0)	23.1 (4.3)	*0.05*
	**(n) %**	**(n) %**	
**Drinking water arsenic quartiles**			
Q4 (Mean: 144.7 μg/L, range: 32–1,400 μg/L)	*(68)* 14.56	*(244)* 30.69	*<0.0001*
Q3 (Mean: 12.0 μg/L, range: 2.1–31 μg/L)	*(77)* 16.49	*(226)* 28.43	
Q2 (Mean: 1.5 μg/L; range: 0.9–2 μg/L	*(185)* 39.61	*(142)* 17.86	
Q1 (Mean: 0.7 μg/L; range: 0.5–0.89 μg/L)	*(137)* 29.34	*(183)* 23.02	
**BMI at time of enrollment (kg/m2)**			
<18.5	*(117)* 25.05	*(246)* 30.94	*0.05*
18.5–24.9	*(299)* 64.03	*(481)* 60.5	
≥ 25.0	*(51)* 10.92	*(68)* 8.55	
**Environmental tobacco smoke**			
Absent	*(319)* 68.6	*(418)* 52.64	*<0.001*
Present	*(146)* 31.4	*(376)* 47.36	
*Missing*	*2*	*1*	
**Maternal education**			
Illiterate/Able to write name	*(47)* 10.13	*(144)* 18.14	*<0.0001*
Primary education	*(199)* 42.89	*(216)* 27.2	
≥ Secondary education	*(218)* 46.98	*(434)* 54.66	
*Missing*	*3*	*1*	
**Parity**			
0	*(214)* 45.82	*(309)* 38.87	*0.01*
1-2	*(213)* 45.61	*(385)* 48.43	
≥3	*(40)* 8.57	*(101)* 12.7	
**Anemia (hemoglobin ≤ 12 g/dL)**			
No	*(327)* 70.32	*(647)* 81.49	*<0.0001*
Yes	*(138)* 29.68	*(147)* 18.51	
*Missing*	*2*	*1*	
**Ferritin**			
Severe deficiency (<20 μg/L)	*(80)* 18.06	*(108)* 14.3	*0.0007*
Mild deficiency (20–40 μg/L)	*(138)* 31.15	*(179)* 23.71	
None (>40 μg/L)	*(225)* 50.79	*(468)* 61.99	
*Missing*	*24*	*40*	
**Sanitation at home**			
Sanitary latrine	*(372)* 79.83	*(469)* 59.14	*<0.0001*
Other	*(94)* 20.17	*(324)* 40.86	
*Missing*	1	2	
**Taking herbs/medication**			
No	*(459)* 98.5	*(719)* 90.44	*<0.0001*
Yes	*(7)* 1.5	*(76)* 9.56	
*Missing*	*1*	*0*	
**Income (taka/month)**			
<3000	*(45)* 9.89	*(176)* 22.34	*<0.0001*
3001–5000	*(270)* 59.34	*(412)* 52.28	
≥ 5000	*(137)* 30.11	*(175)* 22.21	
Refused/don’t know	*(3)* 0.66	*(25)* 3.17	
*Missing*	*12*	*7*	

^a^Wilcoxon test performed on natural log transformed values.

Frequencies of specific symptoms by categories of arsenic exposure and select characteristics are presented in Table 2. Overall, participants who reported nausea/vomiting and abdominal cramping were exposed to higher concentrations of arsenic in their drinking water compared to women who did not report these symptoms. No difference in arsenic exposure was observed between participants who reported cold/flu/respiratory infection or severe headache compared to women who did not report these symptoms. No difference in arsenic exposure was observed between participants who reported vaginal bleeding or swollen ankles. It should be noted that only 12 participants reported vaginal bleeding or swollen ankles resulting in small sample sizes that limited statistical inference (data not shown).

**Table 2:** Associations specific symptoms and select characteristics of 1,262 mothers that were followed throughout pregnancy (2008–2010)

	**Cold/Flu/infection**			**Nausea/vomiting**			**Abdominal cramping**			**Headache**		
	**No *(n = 1035)***	**Yes *(n = 227)***	**p-value**	**No *(n = 762)***	**Yes *(n = 500)***	**p-value**	**No (n = 794 )**	**Yes (n = 468)**	**p-value**	**No (n = 850)**	**Yes (n = 412)**	**p-value**
**Median (IQR) ln arsenic (μg/L)**	0.69 (−0.13, 3.47)	0.53 (−0.15, 2.83)	*0.11*	0.59 (−0.19, 2.91)	1.96 (0.18, 4.11)	*<0.001*	0.59 (−0.17, 2.71)	2.39 (0.18, 4.21)	*<0.001*	0.64 (−0.11, 3.26)	1.02 (−0.15, 3.81)	*0.23*
**Mean (SD) age (yrs)**	22.8 (4.1)	23.1 (4.6)	*0.46*	22.7 (4.1)	23.1 (4.4)	*0.09*	22.8 (4.2)	23 (4.1)	*0.56*	22.9 (4.2)	22.8 (4.1)	*0.73*
	***(n) *%**	***(n) *%**		**(n) %**	**(n) %**		**(n) %**	**(n) %**		**(n) %**	**(n) %**	
**Drinking water arsenic quartiles**												
Q4 (High)	*(264)* 25.51	*(48)* 21.15	*0.26*	*(152)* 19.95	*(160)* 32.0	*<0.001*	*(150)* 18.89	*(162)*34.62	*<0.001*	*(195)* 22.94	*(117)* 28.4	*0.04*
Q3	*(249)* 24.06	*(54)* 23.79		*(163)* 21.39	*(140)* 28.0		*(163)* 20.53	*(140)* 29.91		*(205)* 24.12	*(98)* 23.79	
Q2	*(260)* 25.12	*(67)* 29.52		*(231)* 30.31	*(96)* 19.2		*(262)* 33.0	*(65)* 13.89		*(239)* 28.12	*(88)* 21.36	
Q1 (Low)	*(262)* 25.31	*(58)* 25.55		*(216)* 28.35	*(104)* 20.8		*(219)* 27.58	*(101)* 21.58		*(211)* 24.82	*(109)* 26.46	
**BMI (kg/m2)**												
<18.5	*(295)* 28.5	*(68)* 29.96	*0.07*	*(206)* 27.03	*(157)* 31.4	*0.24*	*(214)* 26395	*(149)* 31.84	*0.10*	*(243)* 28.59	*(120)* 29.13	*0.87*
18.5-24.9	*(651)* 62.9	*(129)* 56.83		*(481)* 63.12	*(299)* 59.8		*(498)* 62.72	*(282)* 60.26		*(529)* 62.24	*(251)* 60.92	
≥ 25.0	*(89)* 8.6	*(30)* 13.22		*(75)* 9.84	*(44)* 8.8		*(82)* 10.33	*(37)* 7.91		*(78)* 9.18	*(41)* 9.95	
**Second hand smoke**												
Absent	*(622)* 60.27	*(115)* 50.66	*0.008*	*(487)* 64.16	*(250)* 50.0	*<0.001*	*(485)* 61.24	*(252)* 53.96	*0.01*	*(524)* 61.79	*(213)* 51.82	*0.0008*
Present	*(410)* 39.73	*(112)* 49.34		*(272)* 35.84	*(250)* 50.0		*(307)* 38.76	*(215)* 46.04		*(324)* 38.21	*(198)* 48.18	
*Num. missing*	*3*	*0*		*3*	*0*		*2*	*1*		*2*	*1*	
**Maternal education**												
Illiterate/able to write name	*(150)* 14.55	*(41)* 18.06	*0.41*	*(88)* 11.59	*(103)* 20.64	*<0.0001*	*(110)* 13.91	*(81)* 17.34	*0.01*	*(122)* 14.4	*(69)* 16.79	*0.02*
Primary education	*(343)* 33.27	*(72)* 31.72		*(295)* 38.87	*(120)* 24.05		*(284)* 35.9	*(131)* 28.05		*(302)* 35.66	*(113)* 27.49	
≥ Secondary education	*(538)* 52.18	*(114)* 50.22		*(376)* 49.54	*(276)* 55.31		*(397)* 50.19	*(255)* 54.6		*(423)* 49.94	*(229)* 55.72	
*Num. missing*	*4*	*0*		*3*	*1*		*3*	*1*		*3*	*1*	
**Parity**												
0	*(430)* 41.55	*(93)* 40.97	*0.98*	*(323)* 42.39	*(200)* 40.0	*0.06*	*(348)* 43.83	*(175)* 37.39	0.06	*(347)* 40.82	*(176)* 42.72	*0.75*
1-2	*(490)* 47.34	*(108)* 47.58		*(367)* 48.16	*(231)* 46.2		*(365)* 45.97	*(233)* 49.79		*(405)* 47.65	*(193)* 46.84	
≥ 3	*(115)* 11.11	*(26)* 11.45		*(72)* 9.45	*(69)* 13.8		*(81)* 10.2	*(60)* 12.82		*(98)* 11.53	*(43)* 10.11	
**Anemia (hemoglobin ≤ 12 g/dL)**												
No	*(802)* 77.71	*(172)* 75.77	*0.53*	*(569)* 74.87	*(405)* 81.16	*0.009*	*(572)* 72.22	*(402)* 86.08	*<0.0001*	*(652)* 76.89	*(322)* 78.35	*0.56*
Yes	*(230)* 22.29	*(55)* 24.23		*(191)* 25.13	*(94)* 18.84		*(220)* 27.78	*(65)* 13.92		*(196)* 23.11	*(89)* 21.65	
*Num. missing*	*3*	*0*		*2*	*1*		*2*	*1*		*2*	*1*	
**Serum ferritin**												
Severe deficiency (<20 μg/L)	*(159)* 16.21	*(29)* 13.36	*0.55*	*(122)* 16.87	*(66)* 13.89	*<0.0001*	*(132)* 17.79	*(56)* 12.28	*0.02*	*(137)* 16.81	*(51)* 13.32	*0.01*
Mild deficiency (20–40 μg/L)	*(260)* 26.5	*(57)* 26.27		*(221)* 30.57	*(96)* 20.21		*(199)* 26.82	*(118)* 25.88		*(230)* 28.22	*(87)* 22.72	
None (>40 μg/L)	*(562)* 57.29	*(131)* 60.37		*(380)* 52.56	*(313)* 65.89		*(411)* 55.39	*(282)* 61.84		*(448)* 54.97	*(245)* 63.97	
*Num. missing*	*54*	*10*		*39*	*25*		*52*	*12*		*35*	*29*	
**Sanitation at home**												
Sanitary latrine	*(715) 69.22*	*(126)* 55.75	*<0.0001*	*(542)* 71.32	*(299)* 59.92	*<0.0001*	*(580)* 73.23	*(261)* 55.89	*<0.0001*	*(601)* 29.04	*(240)* 58.25	*<0.0001*
Other	*(318)* 30.78	*(100)* 44.25		*(218)* 28.68	*(200)* 40.08		*(212)* 26.77	*(206)* 44.11		*(246)* 29.04	*(172)* 41.75	
*Num. missing*	2	1		2	1		2	1		3	0	
**Taking herbs/medication**												
No	*(988)* 95.55	*(190)* 83.7	*<0.0001*	*(739)* 97.11	*(439)* 87.8	*<0.0001*	*(763)* 96.22	*(415)* 88.68	*<0.0001*	*(818)* 96.35	*(360)* 87.38	*<0.0001*
Yes	*(46)* 4.45	*(37)* 16.3		*(22)* 2.89	*(61)* 12.2		*(30)* 3.78	*(53)* 11.32		*(31)* 3.65	*(52)* 12.62	
*Num. missing*	*1*	*0*		*1*	*0*		*1*	*0*		*1*	*0*	
**Income (taka/month)**												
<3000	*(185)* 18.17	*(36)* 16.0	*0.70*	*(111)* 14.84	*(110)* 22.22	*<0.0001*	*(97)* 12.47	*(124)* 26.67	*<0.0001*	*(133)* 15.95	*(88)* 21.52	*0.006*
3001-5000	*(558)* 54.81	*(124)* 55.11		*(422)* 56.42	*(260)* 52.53		*(451)* 57.97	*(231)* 49.68		*(474)* 56.83	*(208)* 50.86	
≥ 5000	*(254)* 24.95	*(58)* 25.78		*(203)* 27.14	*(109)* 22.02		*(215) 27.63*	*(97)* 20.86		*(214)* 25.66	*(98)* 23.96	
Refused/don’t know	*(21)* 2.06	*(7)* 3.11		*(12)* 1.6	*(16)* 3.23		*(15)* 1.93	*(13)* 2.8		*(13)* 1.56	*(15)* 3.67	
*Num. missing*	17	2		14	5		16	3		16	3	

The association between symptoms and quartiles of arsenic exposure from adjusted logistic regression models are presented in Table 3. The odds of a woman reporting any symptom during pregnancy were higher among those participants with the highest arsenic concentration in their drinking water. There was a significant trend for increasing odds of symptoms with increasing arsenic exposure (P for trend < 0.0001). Compared to the lowest quartile of arsenic, the adjusted odds ratio for any symptom during pregnancy was 0.62 (95% CI: 0.44, 0.88) for the second quartile, 1.83 (95% CI: 1.25, 2.69) for the third quartile, and 2.11 (95% CI: 1.42, 3.13) for the fourth quartile. Upon examining individual symptoms, only nausea/vomiting and abdominal cramping showed consistent associations with arsenic exposure. The odds of self-reported nausea/vomiting was 0.98 (95% CI: 0.68, 1.41), 1.52 (95% CI: 1.05, 2.18), and 1.81 (95% CI: 1.26, 2.60) in the second, third and fourth quartile of arsenic relative to the lowest quartile after adjusting for age, body mass index, second-hand tobacco smoke exposure, educational status, parity, anemia, ferritin, medication usage, type of sanitation at home, and household income. A significant increased odds of abdominal cramping, on the other hand, was only observed in the highest arsenic quartile (aOR: 1.62, 95% CI: 1.13, 2.32) compared to the lowest quartile.

**Table 3:** Associations between quartiles of arsenic exposure and odds of self-reported symptoms during pregnancy for women that were followed throughout pregnancy

	**Any symptom**		**Cold/flu/infection**		**Nausea/vomiting**		**Abdominal cramping**		**Headache**	
	**Crude OR**	**aOR**	**Crude OR**	**aOR**	**Crude OR**	**aOR**	**Crude OR**	**aOR**	**Crude OR**	**aOR**
	**(95% CI)**	**(95% CI)**	**(95% CI)**	**(95% CI)**	**(95% CI)**	**(95% CI)**	**(95% CI)**	**(95% CI)**	**(95% CI)**	**(95% CI)**
Water arsenic										
Q4 (High)	2.69 (1.90–3.81)	2.11 (1.42–3.13)	0.82 (0.54–1.25)	0.71 (0.45–1.14)	2.19 (1.58–3.02)	1.81 (1.26–2.60)	2.34 (1.69–3.24)	1.62 (1.13–2.32)	1.16 (0.84–1.61)	1.11 (0.77–1.90)
Q3	2.20 (1.56, 3.09)	1.83 (1.25–2.69)	0.98 (0.65–1.48)	0.88 (0.55–1.39)	1.78 (1.29–2.47)	1.52 (1.05–2.18)	1.86 (1.34–2.58)	1.31 (0.91–1.88)	0.93 (0.66–1.29)	0.86 (0.59–1.25)
Q2	0.58 (0.42–0.78)	0.62 (0.44–0.88)	1.16 (0.79–1.72)	1.30 (0.85–1.99)	0.86 (0.62–1.21)	0.98 (0.68–1.41)	0.54 (0.38–0.77)	0.55 (0.37–0.81)	0.71 (0.51–1.00)	0.77 (0.53–1.10)
Q1 (Low)	1.0 *Ref*	1.0 *Ref*	1.0 *Ref*	1.0 *Ref*	1.0 *Ref*	1.0 *Ref*	1.0 *Ref*	1.0 *Ref*	1.0 *Ref*	1.0 *Ref*
*Test for trend*	*<0.0001*	*<0.0001*	*0.42*	*0.08*	*<0.0001*	*0.001*	*<0.0001*	*<0.0001*	*0.04*	*0.19*

Adjusted models include age, BMI, second-hand smoke exposure, maternal educational status, parity, anemia status, ferritin status, type of sanitation used at home, use of herbal remedy or medication, and household income. The average arsenic concentration and range for each quartile is: Q4 = 144.7 μg/L (32 μg/L – 1,400 μg/L); Q3 = 12.0 μg/L (2.1 μg/L – 31 μg/L); Q2 = 1.5 μg/L (0.9 μg/L – 2.0 μg/L); and Q1 = 0.7 μg/L (0.5 μg/L – 0.89 μg/L).

Table 4 shows the adjusted odds of self-reported symptoms when arsenic exposure was dichotomized below and above 50 μg/L. Among participants whose drinking water contained arsenic greater than or equal to 50 μg/L, odds of reporting any symptom were two-fold higher than participants whose drinking water contained less than 50 μg/L after adjusting for age, BMI, second-hand tobacco exposure, educational status, parity, anemia, ferritin, household sanitation, medication usage, and household income (aOR: 2.11; 95% CI: 1.47, 3.02). The adjusted odds of nausea/vomiting (aOR: 1.47; 95% CI: 1.09, 1.98) and abdominal cramping (aOR: 1.79; 95% CI: 1.31, 2.45) was also significantly higher among participants whose drinking water contained arsenic ≥50 μg/L compared < 50 μg/L.

**Table 4:** Association between arsenic exposure categorized above/below 50 μg/l and self-reported symptoms during pregnancy in 1,262 women recruited in Bangladesh from multiple logistic regression models (2008–2011)

	**Any symptom**		**Cold/flu/infection**		**Nausea/vomiting**		**Abdominal cramping**		**Severe headache**	
	**Crude OR**	**Adjusted OR**	**Crude OR**	**Adjusted OR**	**Crude OR**	**Adjusted OR**	**Crude OR**	**Adjusted OR**	**Crude OR**	**Adjusted OR**
	**(95% CI)**	**(95% CI)**	**(95% CI)**	**(95% CI)**	**(95% CI)**	**(95% CI)**	**(95% CI)**	**(95% CI)**	**(95% CI)**	**(95% CI)**
Water arsenic										
Above 50 μg/L	2.76 (1.98, 3.83)	2.11 (1.47, 3.02)	0.87 (0.60, 1.26)	0.78 (0.52, 1.16)	1.85 (1.40, 2.43)	1.47 (1.09, 1.98)	2.19 (1.63, 2.94)	1.79 (1.31, 2.45)	1.34 (1.01, 1.78)	1.25 (0.92, 1.71)
Below 50 μg/L	1.0	1.0	1.0	1.0	1.0	1.0	1.0	1.0	1.0	1.0

Adjusted models include age, BMI, second-hand smoke exposure, maternal educational status, parity, anemia status, ferritin status, type of sanitation used at home, use of herbal remedy or medication, and household income.

Table 5 shows the adjusted odds of self-reported symptoms when arsenic exposure was dichotomized below and above 10 μg/L. These results were consistent with the previous analyses. The adjusted odds of reporting any symptom was approximately two-fold higher than participants whose drinking water contained less than 50 μg/L (aOR: 2.99; 95% CI: 2.21, 4.04). The adjusted odds of nausea/vomiting (aOR: 1.62; 95% CI: 1.25, 2.10) and abdominal cramping (aOR: 2.39; 95% CI: 1.83, 3.13) was also significantly higher among participants whose drinking water contained arsenic ≥10 μg/L compared to < 10 μg/L. Interestingly, the adjusted odds of cold/flu/infections was significantly lower among participants whose drinking water contained arsenic greater than or equal to 10 μg/L compared to participants whose drinking water contained less than 10 μg/L (aOR: 0.61, 95% CI: 0.43, 0.86).

**Table 5:** Association between arsenic exposure categorized above/below 10 μg/l and self-reported symptoms during pregnancy in 1,262 women recruited in Bangladesh from multiple logistic regression models (2008–2011)

	**Any symptom**		**Cold/Flu/Infection**		**Nausea/Vomiting**		**Abdominal cramping**		**Severe headache**	
	**Crude OR**	**Adjusted OR**	**Crude OR**	**Adjusted OR**	**Crude OR**	**Adjusted OR**	**Crude OR**	**Adjusted OR**	**Crude OR**	**Adjusted OR**
	**(95% CI)**	**(95% CI)**	**(95% CI)**	**(95% CI)**	**(95% CI)**	**(95% CI)**	**(95% CI)**	**(95% CI)**	**(95% CI)**	**(95% CI)**
Water arsenic										
Above 10 μg/L	3.57 (2.73, 4.66)	2.99 (2.21, 4.04)	0.73 (0.54, 0.99)	0.61 (0.43, 0.86)	2.02 (1.60, 2.55)	1.62 (1.25, 2.10)	2.83 (2.20, 3.64)	2.39 (1.83, 3.13)	1.30 (1.02, 1.65)	1.14 (0.87, 1.50)
Below 10 μg/L	1.0	1.0	1.0	1.0	1.0	1.0	1.0	1.0	1.0	1.0

Adjusted models include age, BMI, second-hand smoke exposure, maternal educational status, parity, anemia status, ferritin status, type of sanitation used at home, use of herbal remedy or medication, and household income.

We also examined the potential for effect modification by parity and observed no significant interactions between parity status (primiparous vs multiparous) and arsenic quartile for any symptom (p = 0.78), nausea/vomiting (p = 0.22) or abdominal cramping (p = 0.61). This indicated that the odds of reporting symptoms did not differ between primigravid and multigravid participants in relationship to arsenic exposure.

To determine if taking medication during pregnancy influenced the observed relationship between arsenic and self-reported symptoms during pregnancy, we conducted a restricted analysis (Table 6). In a subset of women who did not report taking any prescription or herbal medications during pregnancy (n = 1,166), we observed similar results where increasing arsenic exposure was significantly associated with increased odds of any symptom (p-value < 0.001), nausea/vomiting (p-value = 0.0007), and abdominal cramping (p-value <0.0001). However, there was no significant trend between arsenic exposure and cold/flu/infection (p-value = 0.39) or severe headaches (p-value = 0.37). Similar associations were also observed when the analysis was restricted to only live births (data not shown).

**Table 6:** Associations between quartiles of arsenic exposure and odds of self-reported symptoms during pregnancy in a subpopulation of women who did not report taking prescription or herbal medication during pregnancy (N = 1,166)

	**Any symptom**		**Cold/flu/infection**		**Nausea/vomiting**		**Abdominal cramping**		**Headache**	
**Water arsenic**	**Crude OR**	**aOR**	**Crude OR**	**aOR**	**Crude OR**	**aOR**	**Crude OR**	**aOR**	**Crude OR**	**aOR**
	**(95% CI)**	**(95% CI)**	**(95% CI)**	**(95% CI)**	**(95% CI)**	**(95% CI)**	**(95% CI)**	**(95% CI)**	**(95% CI)**	**(95% CI)**
Q4 (High)	2.84 (1.98–4.07)	2.33 (1.56–3.50)	0.79 (0.50–1.27)	0.79 (0.47–1.31)	2.17 (1.54–3.06)	1.86 (1.27–2.71)	2.49 (1.77–3.51)	1.80 (1.23–2.62)	1.14 (0.81–1.62)	1.09 (0.74–1.61)
Q3	2.35 (1.66, 3.34)	2.06 (1.39–3.05)	0.96 (0.61–1.51)	0.91 (0.55–1.51)	1.78 (1.27–2.51)	1.55 (1.06–2.27)	1.96 (1.39–2.76)	1.45 (0.99–2.13)	0.97 (0.68–1.38)	0.91 (0.61–1.35)
Q2	0.60 (0.44–0.83)	0.64 (0.45–0.92)	1.30 (0.86–1.98)	1.47 (0.94–2.32)	0.86 (0.61–1.22)	0.94 (0.64–1.37)	0.55 (0.37–0.80)	0.55 (0.37–0.83)	0.72 (0.50–1.02)	0.79 (0.54–1.16)
Q1 (Low)	1.0 *Ref*	1.0 *Ref*	1.0 *Ref*	1.0 *Ref*	1.0 *Ref*	1.0 *Ref*	1.0 *Ref*	1.0 *Ref*	1.0 *Ref*	1.0 *Ref*
Test for trend	<0.0001	<0.0001	0.13	0.39	<0.0001	0.0007	*<0.0001*	*<0.0001*	*0.04*	*0.37*

Adjusted models include age, BMI, second-hand smoke exposure, maternal educational status, parity, anemia status, ferritin status, type of sanitation used at home, use of herbal remedy or medication, and household income. The average arsenic concentration and range for each quartile is: Q4 = 142.8 μg/L (32 μg/L – 1,400 μg/L); Q3 = 11.9 μg/L (2.1 μg/L – 31 μg/L); Q2 = 1.5 μg/L (0.9 μg/L – 2.0 μg/L); and Q1 = 0.7 μg/L (0.5 μg/L – 0.89 μg/L).

## Discussion

We observed that the likelihood that a women reported adverse health symptoms during pregnancy increased with higher arsenic exposure. Specifically, the odds of nausea/vomiting and abdominal cramping were significantly higher among participants in the third quartile and fourth quartile. This is noteworthy because the mean arsenic concentration in the third quartile was 12 μg As/L (range, 2.1-31 μg As/L). Not only is this concentrations of arsenic commonly observed in potable groundwater, this water would be considered safe to drink in Bangladesh because it is below the current recommended level of 50 μg As/L. It is plausible that women whose tubewell met the current recommendations in Bangladesh would be less likely to consider their drinking water to be problematic and subsequently this group may be less inclined to exaggerate symptomology relating to the arsenic levels in their water source compared to participants whose drinking water did not meet the Government’s recommendations.

Nausea/vomiting and abdominal cramping are common ailments in pregnancy and will range in severity [23,24]. While these symptoms tend to resolve during pregnancy and are frequently considered an expected aspect of pregnancy, nausea/vomiting during pregnancy are linked with maternal distress and contribute to maternal morbidity [25-29]. In Sri Lanka, nausea/vomiting have been shown to be the leading cause of lost household productivity among pregnant women [9]. Nausea/vomiting is also highly correlated with maternal anxiety, maternal depression, and contribute to a reduction in maternal quality of life [27,30,31]. Data from studies that examined the relationship between nausea/vomiting and infant birthweight are inconsistent with some studies showing no relationship with birthweight and some reporting that severe nausea/vomiting was associated with decreased birthweight [32,33].

Our finding that arsenic exposure is related to nausea/vomiting during pregnancy increases our understanding of the etiology of this pregnancy-related condition. Studies in other populations also report that nausea/vomiting during pregnancy is more common in younger women, primigravidas, and obese women although these associations were not observed in our population [22]. Researchers have also reported that nausea during pregnancy is related to human chorionic gonadotropin levels, and the absence of these symptoms in early pregnancy may be sign of poor implantation or miscarriage [34]. However, our study includes measures of nausea/vomiting through the second and third trimester which is not likely to represent implantation, an early event in pregnancy. Also, we observed a consistent association between higher levels of arsenic exposure and nausea/vomiting among only those pregnancies that resulted in live birth.

While others have reported that nausea is common in individuals who experienced acute arsenic poisoning [35-37] this study contributes new evidence that chronic arsenic exposure at relatively moderate concentrations are associated with increased risk of nausea [38]. We observe, however, that relatively modest arsenic concentrations in drinking water were associated with increased odds of nausea/vomiting and abdominal cramping during pregnancy. Our study has many strengths including the ascertainment of arsenic exposure early in pregnancy from personal drinking water samples, confirmed singleton pregnancies, and the ability to control for many confounders including second-hand smoke exposure, sanitary conditions, medication usage, and others. Furthermore, our findings remained consistent when we accounted for the potential effect of parity and medication usage. The potential for bias for self-reported maternal symptoms is limited because the prospective analyses yielded very similar results to cross-sectional analyses conducted at the time of enrollment using all available data (Additional file 1: Supplemental Tables S1 and S2). Also, participants were asked three times throughout their pregnancy to report any symptoms. Additionally, participants did not know the concentration of arsenic in their drinking water prior to enrolling in the study. It is possible, however, that participants had previously tested their water for arsenic and this knowledge may have resulted in differential report of maternal symptoms. However, the higher odds of nausea/vomiting were observed at arsenic concentrations below the current Bangladesh drinking water recommendations and subsequently the well would be considered an acceptable source of potable water.

## Conclusion

We observed a positive exposure-response relationship between arsenic concentrations in drinking water and self-reported nausea/vomiting and abdominal cramping during pregnancy in a prospective cohort of pregnant women recruited in Bangladesh. These data provide additional evidence that arsenic-contaminated drinking water at moderate concentrations adversely affects maternal and reproductive health. Continued efforts to reduce arsenic exposure in Bangladesh are warranted particularly for women of reproductive age.

## Abbreviations

BMI: Body mass index; CI: Confidence intervals; GM: Geometric mean; GSD: Geometric standard deviation; LOD: Limit of detection; OR: Odds ratio; WGS: Weeks gestational age; Q: Quartile.

## Competing interests

The authors declare they have no competing interests.

## Authors’ contributions

MLK conceived the hypothesis, supervised data collection and analysis, and developed the manuscript. CBD supervised data entry and contributed to data analysis and manuscript preparation. MM contributed clinical interpretation and manuscript preparation. ER supervised data entry including quality control and contributed to manuscript preparation. ND was responsible for database management. MG supervised field activities and data collection. QQ also supervised data collection and clinical operations in Bangladesh, and contributed to manuscript preparation. MR also supervised field activities and clinical operations in Bangladesh. DCC is the principal investigator of the cohort and contributed to manuscript preparation. All authors contributed equally to the research described in this manuscript. All authors read and approved the final manuscript.

## Supplementary Material

Additional file 1: Table S1 Cross-sectional Bivariate Associations Between Quartiles of Arsenic Exposure and Odds of Self-Reported Symptoms Described At Enrollment in 1,458 Women Recruited Into A Prospective Study in Bangladesh (2008-2010). The average arsenic concentration and range for each quartile is: Q4 = 139.7 μg/L (28.9 μg/L – 1,400 μg/L); Q3 = 10.5 μg/L (2.0 μg/L – 28 μg/L); Q2 = 1.5 μg/L (0.9 μg/L – 1.9 μg/L); and Q1 = 0.71 μg/L (0.5 μg/L – 0.87 μg/L). **Table S2.** Cross-sectional Associations Between Quartiles of Arsenic Exposure and Adjusted Odds of Self-Reported Symptoms Described At Enrollment in 1,347 Pregnant Women Who Participated in A Prospective Study in Bangladesh With Complete Information For Age, BMI, Second-Hand Tobacco Smoke Exposure, Maternal Education, Parity, Anemia Status, Ferritin Status, Use of Herbs or Medication During Pregnancy, Type of Sanitation Used at Home, and Household Income. The average arsenic concentration and range for each quartile is: Q4 = 140.9 μg/L (28.9 μg/L – 1,400 μg/L); Q3 = 10.5 μg/L (2.0 μg/L – 28 μg/L); Q2 = 1.5 μg/L (0.9 μg/L – 1.9 μg/L); and Q1 = 0.7 μg/L (0.5 μg/L – 0.87 μg/L). **Table S3.** Cross-sectional Association Between Arsenic Exposure Categorized Above/Below 50 μg/L and Odds of Self-Reported Symptoms Described At Enrollment. Adjusted Odds Ratios Control For Age, BMI, Second-Hand Tobacco Smoke Exposure, Maternal Education, Parity, Anemia Status, Ferritin Status, Use of Herbs or Medication During Pregnancy, Type of Sanitation Used at Home, and Household Income. **Table S4.** Cross-sectional Associations Between Arsenic Exposure Categorized Above/Below 10 μg/L and Odds of Self-Reported Symptoms Described At Enrollment. Adjusted Odds Ratios Control For Age, BMI, Second-Hand Tobacco Smoke Exposure, Maternal Education, Parity, Anemia Status, Ferritin Status, Use of Herbs or Medication During Pregnancy, Type of Sanitation Used at Home, and Household Income. Click here for file
