# Supplementary material for: A prospective cohort study of the association between drinking water arsenic exposure and self-reported maternal health symptoms during pregnancy in Bangladesh
Source: Environ Health. 2014 Apr 16;13:29. doi: 10.1186/1476-069X-13-29 (PMC4021291; doi:10.1186/1476-069X-13-29)
Supplement: Additional file 1: Table S1 — Cross-sectional Bivariate Associations Between Quartiles of Arsenic Exposure and Odds of Self-Reported Symptoms Described At Enrollment in 1,458 Women Recruited Into A Prospective Study in Bangladesh (2008-2010). The average arsenic concentration and range for each quartile is: Q4 = 139.7 μg/L (28.9 μg/L – 1,400 μg/L); Q3 = 10.5 μg/L (2.0 μg/L – 28 μg/L); Q2 = 1.5 μg/L (0.9 μg/L – 1.9 μg/L); and Q1 = 0.71 μg/L (0.5 μg/L – 0.87 μg/L). Table S2. Cross-sectional Associations Between Quartiles of Arsenic Exposure and Adjusted Odds of Self-Reported Symptoms Described At Enrollment in 1,347 Pregnant Women Who Participated in A Prospective Study in Bangladesh With Complete Information For Age, BMI, Second-Hand Tobacco Smoke Exposure, Maternal Education, Parity, Anemia Status, Ferritin Status, Use of Herbs or Medication During Pregnancy, Type of Sanitation Used at Home, and Household Income. The average arsenic concentration and range for each quartile is: Q4 = 140.9 μg/L (28.9 μg/L – 1,400 μg/L); Q3 = 10.5 μg/L (2.0 μg/L – 28 μg/L); Q2 = 1.5 μg/L (0.9 μg/L – 1.9 μg/L); and Q1 = 0.7 μg/L (0.5 μg/L – 0.87 μg/L). Table S3. Cross-sectional Association Between Arsenic Exposure Categorized Above/Below 50 μg/L and Odds of Self-Reported Symptoms Described At Enrollment. Adjusted Odds Ratios Control For Age, BMI, Second-Hand Tobacco Smoke Exposure, Maternal Education, Parity, Anemia Status, Ferritin Status, Use of Herbs or Medication During Pregnancy, Type of Sanitation Used at Home, and Household Income. Table S4. Cross-sectional Associations Between Arsenic Exposure Categorized Above/Below 10 μg/L and Odds of Self-Reported Symptoms Described At Enrollment. Adjusted Odds Ratios Control For Age, BMI, Second-Hand Tobacco Smoke Exposure, Maternal Education, Parity, Anemia Status, Ferritin Status, Use of Herbs or Medication During Pregnancy, Type of Sanitation Used at Home, and Household Income. [file 1476-069X-13-29-S1.docx]

**Table S1.** Cross-sectional Bivariate Associations Between Quartiles of Arsenic Exposure and Odds of Self-Reported Symptoms Described At Enrollment in 1,458 Women Recruited Into A Prospective Study in Bangladesh (2008-2010). The average arsenic concentration and range for each quartile is: Q4 = 139.7 µg/L (28.9 µg/L – 1,400 µg/L); Q3 = 10.5 µg/L (2.0 µg/L – 28 µg/L); Q2 = 1.5 µg/L (0.9 µg/L – 1.9 µg/L); and Q1 = 0.71 µg/L (0.5 µg/L – 0.87 µg/L).

|  | Any Symptom | | | Cold/Flu/Infection | | | Nausea/Vomiting | | | Abdominal Cramping | | | Headache | | |
| --- | --- | --- | --- | --- | --- | --- | --- | --- | --- | --- | --- | --- | --- | --- | --- |
| Water Arsenic | No | Yes | Crude OR  (95% CI) | No | Yes | Crude OR  (95% CI) | No | Yes | Crude OR  (95% CI) | No | Yes | Crude OR (95% CI) | No | Yes | Crude OR  (95% CI) |
| Q4 (High) | 107 | 257 | 2.29 (1.69, 3.10) | 318 | 46 | 0.86 (0.56, 1.31) | 183 | 181 | 2.40 (1.77, 3.26) | 221 | 143 | 1.55 (1.14, 2.11) | 242 | 122 | 1.19 (0.87, 1.63) |
| Q3 | 125 | 231 | 1.76 (1.31, 2.37) | 306 | 50 | 0.97 (0.64, 1.47) | 199 | 157 | 1.92 (1.41, 2.61) | 226 | 130 | 1.38 (1.01, 1.88) | 247 | 109 | 1.05 (0.76, 1.44) |
| Q2 | 221 | 150 | 0.65 (0.48, 0.87) | 305 | 66 | 1.28 (0.86, 1.90) | 269 | 102 | 0.92 (0.67, 1.27) | 299 | 72 | 0.58 (0.41, 0.81) | 280 | 91 | 0.77 (0.56, 1.07) |
| Q1 (Low) | 179 | 188 | 1.0 *Ref* | 314 | 53 | 1.0 *Ref* | 260 | 107 | 1.0 *Ref* | 259 | 108 | 1.0 *Ref* | 258 | 109 | 1.0 *Ref* |
| *Test for Trend* |  |  | *<0.0001* |  |  | *0.24* |  |  | *<0.0001* |  |  | *<0.0001* |  |  | *0.06* |

**Table S2.** Cross-sectional Associations Between Quartiles of Arsenic Exposure and Adjusted Odds of Self-Reported Symptoms Described At Enrollment in 1,347 Pregnant Women Who Participated in A Prospective Study in Bangladesh With Complete Information For Age, BMI, Second-Hand Tobacco Smoke Exposure, Maternal Education, Parity, Anemia Status, Ferritin Status, Use of Herbs or Medication During Pregnancy, Type of Sanitation Used at Home, and Household Income. The average arsenic concentration and range for each quartile is: Q4 = 140.9 µg/L (28.9 µg/L – 1,400 µg/L); Q3 = 10.5 µg/L (2.0 µg/L – 28 µg/L); Q2 = 1.5 µg/L (0.9 µg/L – 1.9 µg/L); and Q1 = 0.7 µg/L (0.5 µg/L – 0.87 µg/L).

|  | Any symptom | | | Cold/Flu/Infection | | | Nausea/Vomiting | | | Abdominal cramping | | | Headache | | |
| --- | --- | --- | --- | --- | --- | --- | --- | --- | --- | --- | --- | --- | --- | --- | --- |
| Water Arsenic | No | Yes | aOR  (95% CI) | No | Yes | aOR  (95% CI) | No | Yes | aOR  (95% CI) | No | Yes | aOR  (95% CI) | No | Yes | aOR (95% CI) |
| Q4 (High) | 102 | 244 | 1.83 (1.31, 2.57) | 303 | 43 | 0.69 (0.42, 1.12) | 175 | 171 | 1.91 (1.35, 2.68) | 208 | 138 | 1.11 (0.79, 1.57) | 229 | 117 | 1.04 (0.73, 1.49) |
| Q3 | 118 | 222 | 1.52 (1.09, 2.12) | 291 | 49 | 0.90 (0.56, 1.43) | 189 | 151 | 1.62 (1.15, 2.28) | 214 | 126 | 1.05 (0.75, 1.49) | 238 | 102 | 0.92 (0.64, 1.31) |
| Q2 | 202 | 137 | 0.71 (0.51, 0.97) | 279 | 60 | 1.38 (0.90, 2.14) | 242 | 97 | 1.04 (0.73, 1.47) | 272 | 67 | 0.59 (0.41, 0.85) | 256 | 83 | 0.84 (0.59, 1.21) |
| Q1 (Low) | 159 | 163 | 1.01.0 *Ref* | 276 | 46 | 1.0 *Ref* | 228 | 94 | 1.0 *Ref* | 225 | 97 | 1.0 *Ref* | 230 | 92 | 1.0 *Ref* |
| *Test for Trend* |  |  | *<0.0001* |  |  | *0.03* |  |  | *0.0002* |  |  | *0.004* |  |  | *0.71* |

**Table S3.** Cross-sectional Association Between Arsenic Exposure Categorized Above/Below 50 µg/L and Odds of Self-Reported Symptoms Described At Enrollment. Adjusted Odds Ratios Control For Age, BMI, Second-Hand Tobacco Smoke Exposure, Maternal Education, Parity, Anemia Status, Ferritin Status, Use of Herbs or Medication During Pregnancy, Type of Sanitation Used at Home, and Household Income.

|  | Any symptom | | Cold/Flu/Infection | | Nausea/Vomiting | | Abdominal cramping | | Headache | |
| --- | --- | --- | --- | --- | --- | --- | --- | --- | --- | --- |
|  | Crude OR (95% CI) | aOR (95% CI) | Crude OR (95% CI) | aOR (95% CI) | Crude OR (95% CI) | aOR (95% CI) | Crude OR (95% CI) | aOR (95% CI) | Crude OR (95% CI) | aOR (95% CI) |
| ≥50 µg As/L | 2.59 (1.94, 3.47) | 2.12 (1.55, 2.89) | 0.88 (0.60, 1.28) | 0.78 (0.50, 1.16) | 1.97 (1.51, 2.56) | 1.55 (1.17, 2.06) | 1.71 (1.31, 2.23) | 1.35 (1.01, 1.81) | 1.35 (1.02, 1.78) | 1.24 (0.92, 1.68) |
| < 50 µg As/L | 1.0 *Ref* | 1.0 *Ref* | 1.0 *Ref* | 1.0 *Ref* | 1.0 *Ref* | 1.0 *Ref* | 1.0 *Ref* | 1.0 *Ref* | 1.0 *Ref* | 1.0 *Reference* |

**Table S4.** Cross-sectional Associations Between Arsenic Exposure Categorized Above/Below 10 µg/L and Odds of Self-Reported Symptoms Described At Enrollment. Adjusted Odds Ratios Control For Age, BMI, Second-Hand Tobacco Smoke Exposure, Maternal Education, Parity, Anemia Status, Ferritin Status, Use of Herbs or Medication During Pregnancy, Type of Sanitation Used at Home, and Household Income.

|  | Any symptom | | Cold/Flu/Infection | | Nausea/Vomiting | | Abdominal cramping | | Headache | |
| --- | --- | --- | --- | --- | --- | --- | --- | --- | --- | --- |
|  | Crude OR (95% CI) | aOR (95% CI) | Crude OR (95% CI) | aOR (95% CI) | Crude OR (95% CI) | aOR (95% CI) | Crude OR (95% CI) | aOR (95% CI) | Crude OR (95% CI) | aOR (95% CI) |
| ≥10 µg As/L | 2.71 (2.16, 3.42) | 2.53 (1.75, 2.91) | 0.65 (0.47, 0.89) | 0.51 (0.35, 0.74) | 2.14 (1.71, 2.66) | 1.67 (1.30, 2.14) | 1.91 (1.52, 2.40) | 1.46 (1.13, 1.88) | 1.24 (0.98, 1.57) | 1.07 (0.82, 1.40) |
| <10 µg As/L | 1.0 *Ref* | 1.0 *Ref* | 1.0 *Ref* | 1.0 *Ref* | 1.0 *Ref* | 1.0 *Ref* | 1.0 *Ref* | 1.0 *Ref* | 1.0 *Ref* | 1.0 *Ref* |
